# Alzheimer’s disease Advax^CpG^- adjuvanted MultiTEP-based dual and single vaccines induce high-titer antibodies against various forms of tau and Aβ pathological molecules

**DOI:** 10.1038/srep28912

**Published:** 2016-07-01

**Authors:** Hayk Davtyan, Karen Zagorski, Harinda Rajapaksha, Armine Hovakimyan, Arpine Davtyan, Irina Petrushina, Konstantin Kazarian, David H. Cribbs, Nikolai Petrovsky, Michael G. Agadjanyan, Anahit Ghochikyan

**Affiliations:** 1 Department of Molecular Immunology, Institute for Molecular Medicine, Huntington Beach, California, 92647, USA; 2 Vaxine Pty Ltd, Flinders Medical Center, Bedford Park, Adelaide, 5042, Australia; 3 Department of Diabetes and Endocrinology, Faculty of Medicine, Flinders University, Adelaide, 5042, Australia; 4 The Institute for Memory Impairments and Neurological Disorders, University of California, Irvine, Irvine, California, 92697, USA; 5 Department of Neurology, University of California Irvine, Irvine, California, 92697, USA

## Abstract

Although β-amyloid (Aβ) may be the primary driver of Alzheimer’s disease (AD) pathology, accumulation of pathological tau correlates with dementia in AD patients. Thus, the prevention/inhibition of AD may require vaccine/s targeting Aβ and tau simultaneously or sequentially. Since high antibody titers are required for AD vaccine efficacy, we have decided to generate vaccines, targeting Aβ (AV-1959R), Tau (AV-1980R) or Aβ/tau (AV-1953R) B cell epitopes, based on immunogenic MultiTEP platform and evaluate the immunogenicity of these vaccines formulated with Advax^CpG^, delta inulin, Alhydrogel^®^, Montanide-*ISA51*, Montanide-*ISA720*, MPLA-SM pharmaceutical grade adjuvants. Formulation of AV-1959R in Advax^CpG^ induced the highest cellular and humoral immune responses in mice. The dual-epitope vaccine, AV-1953R, or the combination of AV-1959R and AV-1980R vaccines formulated with Advax^CpG^ induced robust antibody responses against various forms of both, Aβ and tau pathological molecules. While anti-Aβ antibody titers after AV-1953R immunization were similar to that in mice vaccinated with AV-1959R or AV-1959R/AV-1980R combination, anti-tau titers were significantly lower after AV-1953R injection when compared to the AV-1980R or AV-1959R/AV-1980R. *In silico* 3D-modeling provided insight into the differences in immunogenicity of these vaccine constructs. In sum, AV-1959R and AV-1980R formulated with Advax^CpG^ adjuvant were identified as promising immunogenic vaccines for ongoing pre-clinical assessment and future human clinical trials.

Aβ-mediated plaque formation is thought to be the primary event in Alzheimer’s disease (AD) pathogenesis^1^,^2^,^3^. Later, AD pathology becomes self-propagating^4^,^5^,^6^,^7^ with less dependence on Aβ and greater involvement of other proteins such as tau^8^. The temporal relationship of misfolded proteins in AD pathogenesis may have relevance to AD vaccine strategies. Hence, vaccines targeting Aβ only may be effective prior to or in the very early stages of AD pathogenesis, whereas vaccines targeting tau may remain effective in latter stages of AD. Therefore, the most effective strategy may be to develop an immunogenic vaccine or vaccines targeting both Aβ and tau, such that the same vaccine or the combination of vaccines would then be effective across the entire spectra of AD progression.

Safety is an important consideration in AD vaccine development given cases of aseptic meningoencephalitis observed previously in the AN-1792 clinical trials and likely associated with autoreactive T cell infiltration into the brains of vaccinated subjects^9^. To avoid this risk, we have developed the MultiTEP vaccine platform that consists of a string of 12 non-self, pathogen-derived T helper (Th) epitopes^10^, to which we can attach different B cell self-epitopes from neuronal proteins involved in AD pathogenesis. Previously we have demonstrated that a DNA vaccine composed of three copies of a B cell epitope from the N-terminal region of Aβ (Aβ_1-11_) attached to the MultiTEP protein (AV-1959D) was highly immunogenic in mice^10^,^11^, rabbits^12^ and macaques^10^,^13^. To develop a vaccine targeting pathological tau we decided to use the same immunogenic MultiTEP platform incorporating the tau_2-18_ epitope. We chose this epitope because it was previously shown that tau_2-18_ is normally hidden in microtubule bound tau conformation but becomes highly exposed during tau aggregation^14^,^15^. Importantly, this region of tau, also termed the phosphatase-activating domain (PAD), plays an important role in activation of a signaling cascade involving PP1 and GSK-3 that leads to dissociation of cargo from kinesins and therefore anterograde fast axonal transport (FAT) inhibition. The exposure of PAD that is required for inhibition of FAT might be regulated by PAD phosphorylation, as well as by the N-terminal truncation of tau that occurs during neurofibrillary tangle formation. Phosphorylation of Y18 as well as truncation of the N-terminal region of aggregated tau has been suggested to remove the toxic region and have a protective role^14^,^15^,^16^,^17^. Thus, we hypothesized that anti-tau_2-18_ antibodies will preferentially recognize pathological rather than normal forms of tau, and thereby prevent its aggregation and PAD mediated toxicity during the early stages of tauopathy.

Here we describe for the first time the generation of MutiTEP platform-based recombinant vaccines targeting Aβ_1-11_, (AV-1959R), tau_2-18_ (AV-1980R), or tau_2-18_ and Aβ_1-11_ simultaneously (dual specificity, AV-1953R) and report on the immunogenicity of these vaccines. We also identify a novel adjuvant, Advax^CpG^ derived from delta inulin^18^, that provides optimal immune enhancement for the MutiTEP vaccines.

## Results

### Selection of an optimal adjuvant for anti-Aβ vaccine, AV-1959R

Data from previous clinical trials showed that high anti-Aβ antibody titers correlated with a reduction in brain pathology in AN-1792 immunized AD patients that later came to autopsy, suggesting that therapeutic benefit was closely linked to antibody titers^9^. The cGMP grade delta inulin-based adjuvants, Advax™ and Advax^CpG^ were previously reported to enhance the immunogenicity and efficacy of various vaccines targeting viral and bacterial antigens in pre-clinical studies^18^,^19^,^20^,^21^,^22^ and clinical trials^23^,^24^. To select an adjuvant that will induce the highest antibody response and lowest variability of antibody levels in response to vaccinations of mice with AV-1959R, we tested these adjuvants in parallel with Quil-A^25^, a less purified version of QS21, the adjuvant that was used in the AN-1792 clinical trials^9^ as well as the commercial adjuvants Alhydrogel^®^, Montanide-*ISA51*, Montanide-*ISA720*, and MPLA-SM.

**Figure 1:**
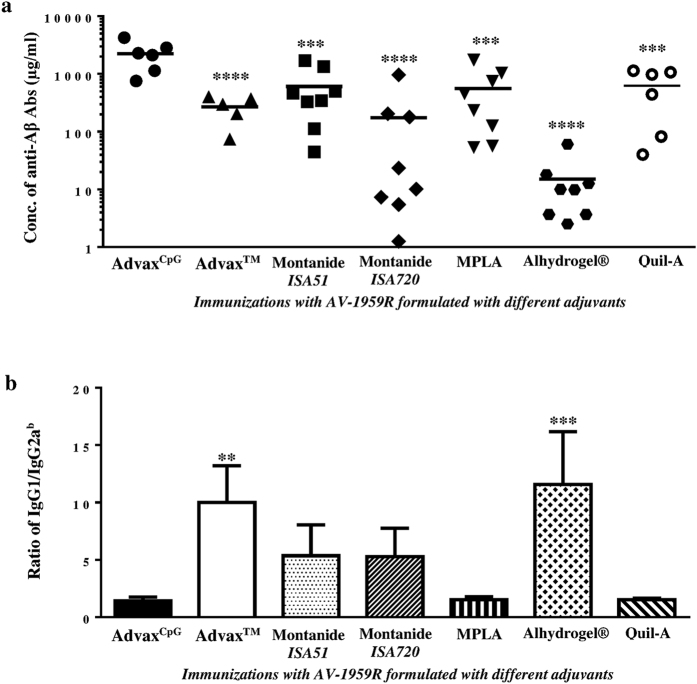
Humoral immune responses in mice vaccinated with AV-1959R protein formulated with cGMP grade adjuvants (Advax^CpG^, Advax^TM^, Montanide-*ISA51*, Montanide-*ISA720*, MPLA, Alhydrogel®) and control adjuvant, Quil-A. (**a**) Concentrations of anti-Aβ antibodies were measured by ELISA in sera collected after the 3^rd^ immunization. Lines represent mean values. (**b**) Isotypes of generated anti-Aβ antibodies had been determined by ELISA (see Fig. S1) and IgG1/IgG2a^b^ ratio was calculated. Bars represent average ± SD (n = 6–8/per group). Statistical significance was calculated against group of mice immunized with AV-1959R formulated in Advax^CpG^ using ANOVA test (**P < 0.01***P < 0.001 *and* ****P < 0.0001).

**Figure 2:**
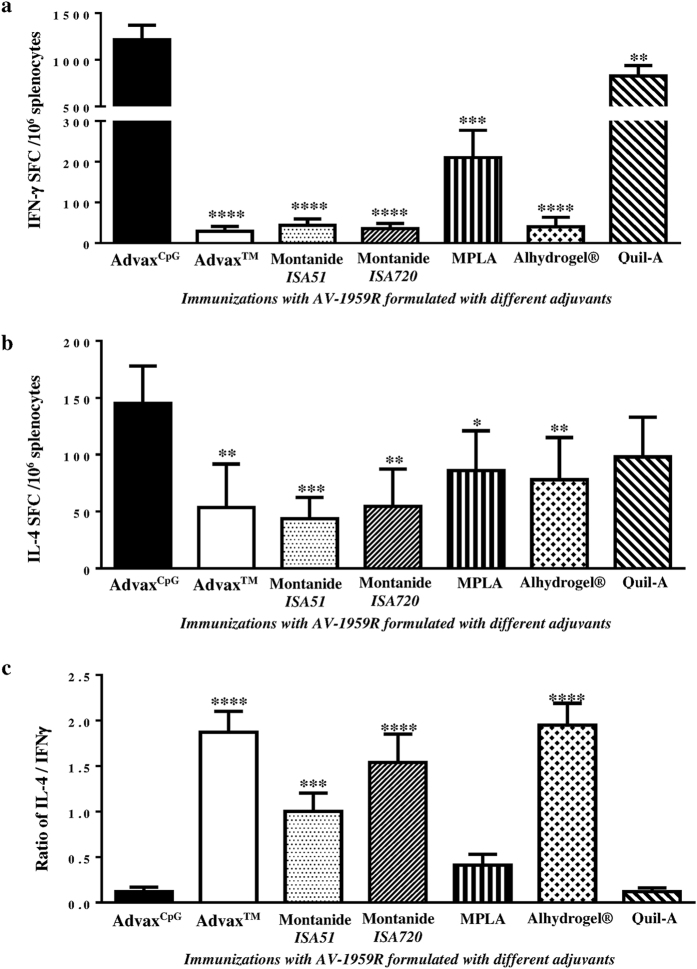
Cellular immune responses in mice vaccinated with AV-1959R protein formulated with cGMP grade adjuvants (Advax^CpG^, Advax^TM^, Montanide-*ISA51*, Montanide-*ISA720*, MPLA, Alhydrogel®) and control adjuvant, Quil-A. (**a,b**) Numbers of IFN-γ (**a**) and IL-4 (**b**) producing T cells were calculated by ELISpot in splenocyte cultures obtained from experimental and control animals. (**c**) IL-4/IFN-γ ratios were calculated based on data presented in (**a,b**). Bars represent average ± SD (n = 6–8/per group). Statistical significances were calculated against group of mice immunized with AV-1959R formulated in Advax^CpG^ using ANOVA test (*P < 0.05, **P < 0.01, ***P < 0.001 *and* ****P < 0.0001).

The results showed that AV-1959R formulated with Advax^CpG^ induced significantly stronger antibody responses than all the other adjuvants with a low variability in responses between animals in the Advax^CpG^ group (Fig. 1a). Analysis of antibody isotypes specific for Aβ showed that Alhydrogel^®^, Advax^TM^, Montanide-*ISA51* and -*ISA720* adjuvants induced primarily an IgG1 (Th2) response, whereas Advax^CpG^, MPLA and Quil-A shifted the response toward IgG2a^b^, a Th1 response associated isotype (Figs 1b and S1). To further explore adjuvant effects on Th1 and Th2 phenotype, we measured the numbers of splenocytes producing IFN-γ and IL-4 cytokines by ELISpot (spot-forming cells, SFC) and found that the Advax^CpG^ group produced significantly higher frequencies of IFN-γ^+^ and IL-4^+^ Th cells than the other adjuvant groups (Fig. 2a,b). The TLR4 agonist, MPLA was the only other GMP-grade adjuvant that generated significant numbers of both IFN-γ^+^ and IL-4^+^ Th cells, although these were approximately 5 and 1.5 times, respectively, lower than those induced with Advax^CpG^ (Fig. 2a,b). Calculation of the ratio of IL-4/IFN-γ positive Th cells (Fig. 2c) supported the antibody isotypes data and confirmed that Advax^CpG^ was the strongest combined Th1 and Th2 adjuvant followed by MPLA, while other adjuvants only generated primarily Th2 responses to immunizations with AV-1959R. The level of Th1 responses induced by the control adjuvant, Quil-A were comparable to MPLA, but significantly lower than in Advax^CpG^ (Figs 1 and 2). Finally, Advax^CpG^ was also well tolerated by all animals with no evidence of either local or systemic vaccine adverse reactions. On the basis of these data, Advax^CpG^ was selected as the sole adjuvant for all subsequent studies with MultiTEP-based AD vaccines targeting Aβ, tau, or Aβ/tau simultaneously or sequentially.

### Immunogenic efficacy of different AD vaccines formulated with Advax^CpG^ adjuvant

**Figure 3:**
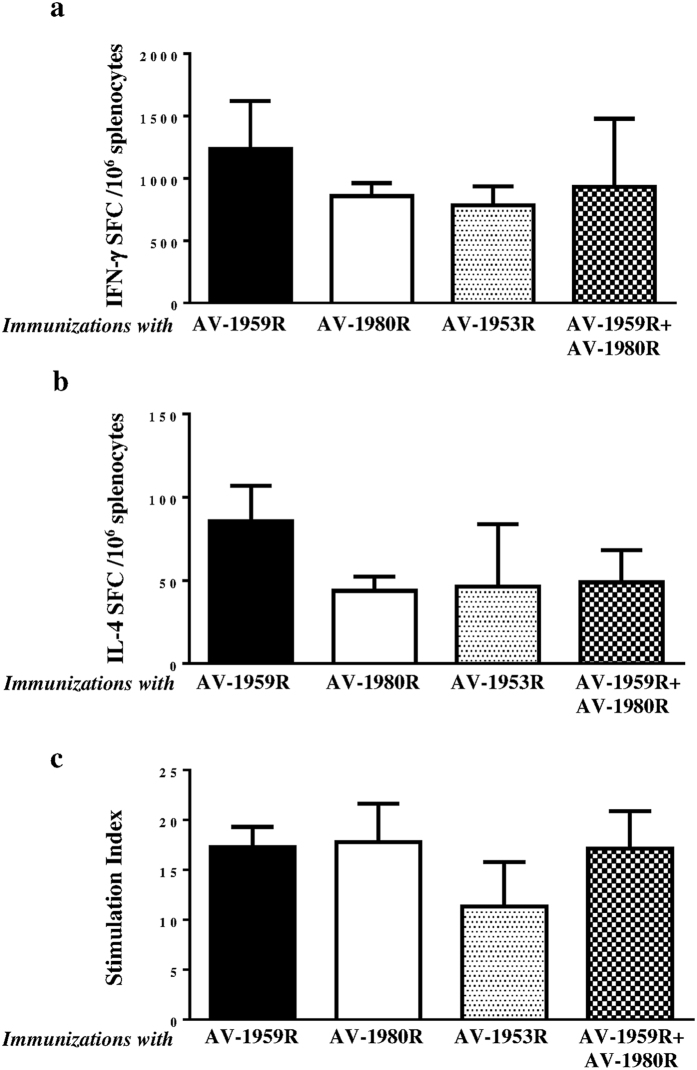
Cellular immune responses in mice immunized with epitope vaccines targeting Aβ (AV-1959R), tau (AV-1980R), and Aβ/tau (dual-epitope vaccine: AV-1953R or mixture of AV-1959R and AV-1980R). Numbers of IFN-γ (**a**) and IL-4 (**b**) producing T cells were calculated by ELISpot in splenocyte cultures obtained from experimental and control animals. (**c**) Proliferation of cells was detected by [3H]-thymidine incorporation assay in the same splenocyte cultures and expressed as stimulation index. Cellular immune responses in control group were at the background level (INF-γ^+^ and IL-4^+^ SFCs were <15, and stimulation index was <1.6). Bars represent average ± SD (n = 8 per group).

**Figure 4:**
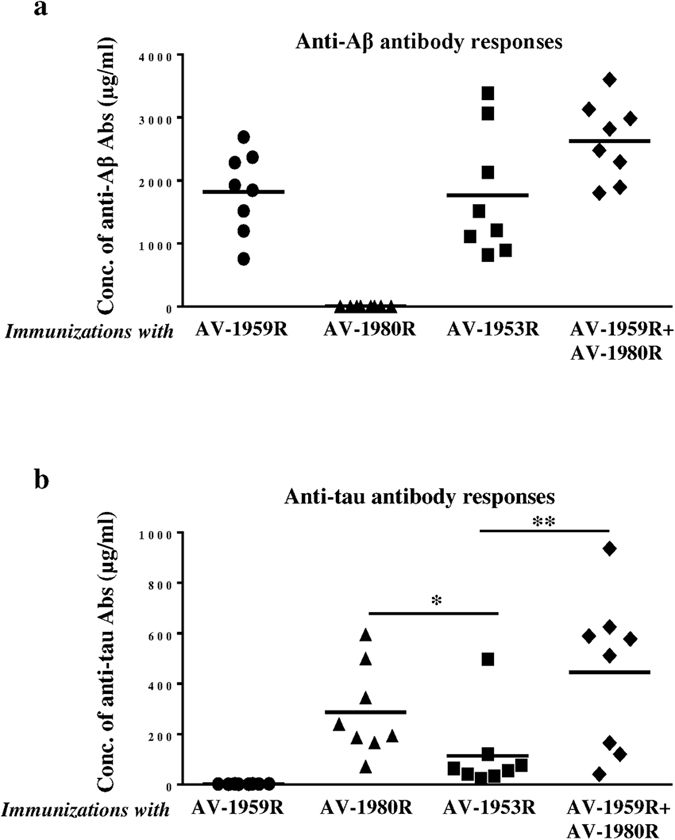
Humoral immune responses in mice vaccinated with AV-1959R, AV-1980R, AV-1953R and mixture of AV-1959R and AV-1980R formulated with Advax^CpG^ adjuvant. Concentrations of anti-Aβ (**a**) and anti-tau (**b**) antibodies were measured by ELISA in sera collected after the 3^rd^ immunization and calculated using calibration curves generated with 6E10 and 1C9 monoclonal antibodies, respectively. Lines represent mean values for n = 8/per group (*P < 0.05, **P < 0.01, ANOVA test).

**Figure 5:**
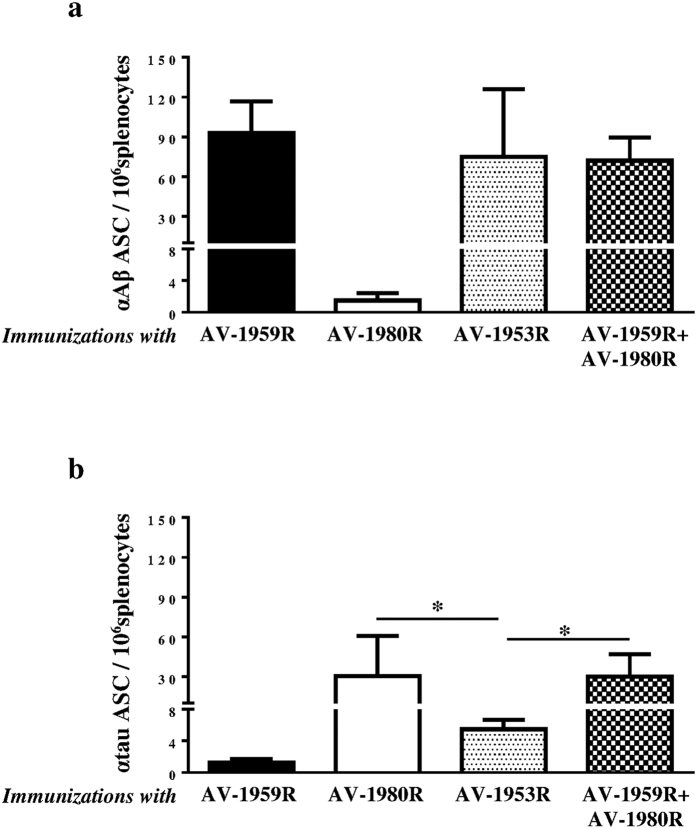
Number of B cells producing anti-Aβ and anti-Tau antibodies in mice vaccinated with AV-1959R, AV-1980R, AV-1953R and mixture of AV-1959R and AV-1980R formulated with Advax^CpG^ adjuvant. Detection of anti-Aβ (**a**) and anti-tau (**b**) antibody-secreting cells (ASC), visualized as spots, was done in splenocyte cultures obtained from experimental and control mice using ELISpot assay. Bars represent average ± SD (n = 8/per group, *P < 0.05, ANOVA test).

All tested AD vaccines formulated with Advax^CpG^ adjuvant generated equally strong T cell responses, measured by detection of IFN-γ^+^, IL4^+^ SFC or splenocytes proliferation specific to foreign Th cell epitopes incorporated in the MultiTEP platform (Fig. 3). Of note, no potentially harmful autoreactive Th cells were detected after re-stimulation of immune splenocytes with Aβ or tau self-epitopes, either by ELISpot or splenocytes proliferation assay (data not shown). Importantly, generation of strong cellular immune responses to Th epitopes incorporated in the MultiTEP vaccine platform supported the production of equally high concentrations of anti-Aβ antibodies in mice vaccinated with AV1959R/AV-1980R combination, AV-1959R, or AV-1953R. As expected, immunization with AV-1980R did not generate anti-Aβ antibodies (Fig. 4a). It should be mentioned that concentrations of anti-tau antibodies were significantly lower in mice immunized with AV-1953R compared to mice vaccinated with the AV-1959R/AV-1980R combination or AV-1980R alone (Fig. 4b). These antibody response patterns were mirrored by the frequency of antibody secreting B cells (ASC); numbers of anti-Aβ ASC were similar in mice immunized with single or combined vaccine formulations, while the numbers of anti-tau ASC were significantly lower in mice vaccinated with the dual-epitope AV-1953R vaccine (Fig. 5).

**Figure 6:**
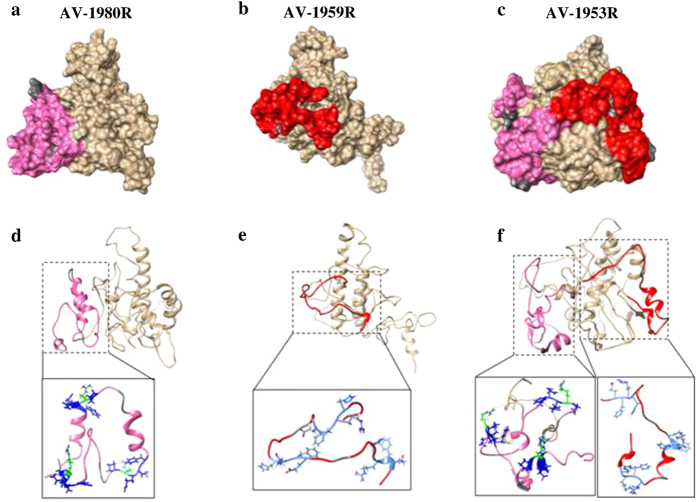
3D structural models of AV-1980R, AV-1959R and AV-1953R synthetic proteins. The surface filled representations of the AV-1980R (**a**), AV-1959R (**b**) and AV1953R (**c**) are presented in the upper panel. Tau and Aβ epitopes on the MultiTEP protein are highlighted in pink and red, respectively. The GS linker is highlighted in dark grey. In the lower panel, critical residues on the AV-1980R epitope (PRQEF) are highlighted in blue (**d**) and the critical residues on the AV-1959R epitope (EFRH) are highlighted in cyan (**e**). In AV-1953R critical residues on each epitope follows AV-1980R and AV-1959R color cording (**f**).

We hypothesized that anti-tau B cell epitopes attached to MultiTEP may not be well presented on the surface of the dual-epitope AV-1953R vaccine compared with the single epitope constructs. To address this possibility we undertook *in silico* structural modeling of the MultiTEP platform-based AV-1980R, AV-1959R and AV-1953R vaccines to examine the surface accessibility of B cell antigenic determinants of Aβ_1-11_ (EFRH)^26^ in AV-1959R and AV-1953R and Tau_2-18_ (PRQEF; paper in preparation) in AV1980R and AV-1953R (Fig. 6). The modeling data suggested that on AV-1980R, two of three tau epitopes are linear with the side chains of the critical amino acid residues accessible on the surface (Fig. 6a,d), while in AV-1959R, all three Aβ epitopes are linear with the side chains of the critical amino acid residues accessible on the surface (Fig. 6b,e). On AV-1953R, two out of three Aβ and two out of three tau epitopes are linear, however, only side chains of Aβ, but not critical tau amino acid residues are easily accessible (Fig. 6c,f). Hence, changes in the epitope structure in combination with alterations in the side chain accessibility of critical residues in the epitopes may have led to the reduced anti-tau immunogenicity of the AV-1953R dual-epitope construct.

### Immune sera recognize various pathological forms of Aβ and tau molecules in AD brains

**Figure 7:**
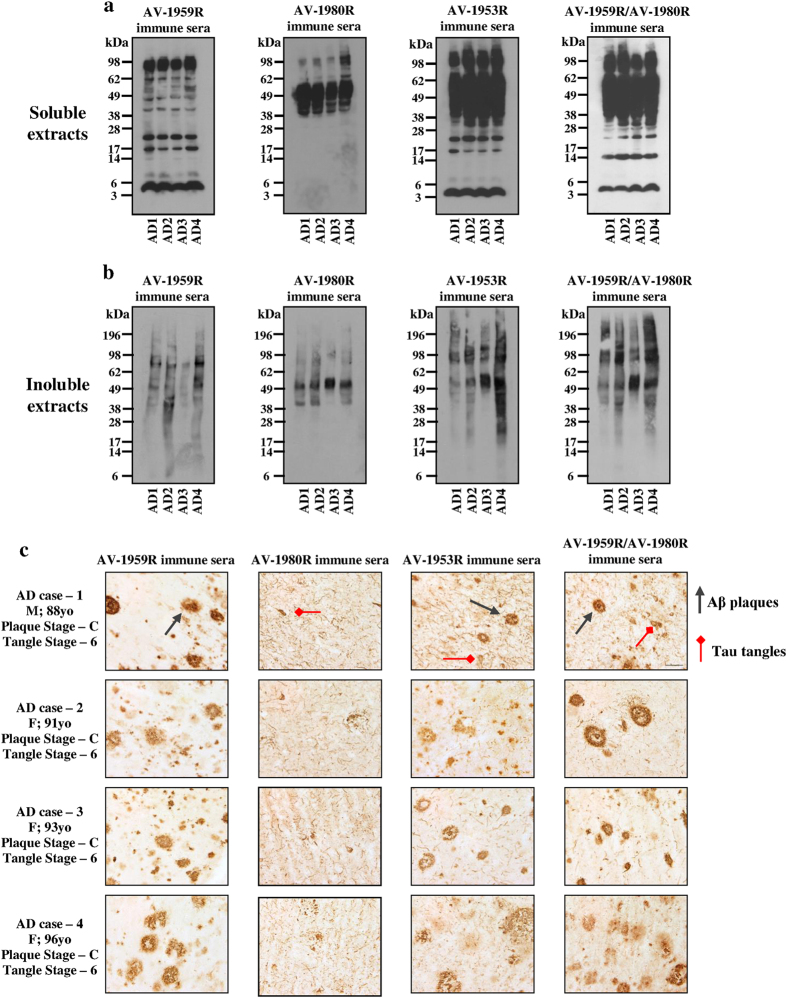
Immune sera isolated from mice vaccinated with AV-1959R, AV-1980R, AV-1953R and mixture of AV-1959R and AV-1980R formulated with Advax^CpG^ adjuvant bound to different forms of Aβ and tau in the brains from AD cases. Western blots of soluble **(a)** and insoluble fractions **(b)** of brain homogenates containing 50 μg total protein from four AD cases were stained with immune sera normalized to 1 μg/ml for anti-Aβ and 0.4 μg/ml for anti-tau antibodies based on ELISA data. **(c)** Immune sera were screened for the ability to bind to human Aβ plaques or/and tau tangles using 40 μm brain sections of formalin-fixed cortical tissue from the same AD cases. The original magnification is 60× and the scale bar is 20 μm.

To demonstrate the effectiveness of antibodies generated in mice immunized with single vaccines, AV-1959R or AV-1980R, the mixture of two vaccines (AV-1959R/AV-1980R) or dual vaccine (AV-1953R), we analyzed the binding of immune sera to various pathological forms of Aβ and Tau in brain tissues from four different AD cases by Western Blot (WB) (Fig. 7a,b) and immunohistochemistry (IHC) (Fig. 7c).

The AV-1959R-immune sera bound monomeric Aβ in soluble as well as low and high molecular weight oligomers in both soluble and insoluble fractions of brain homogenates. As expected, AV-1980R-immune sera recognized monomeric tau as well as multiple larger and smaller species of tau in both soluble and insoluble fractions of brain homogenates. What is more important, antibodies generated by either the mixture of vaccines (AV-1959R/AV-1980R) or the dual vaccine (AV-1953R) recognized the same species of Aβ and tau that were detected by antisera isolated from mice vaccinated with appropriate single vaccines (AV-1959R and AV-1980R). Similar results have been obtained by IHC analyses of the same brain tissues (Fig. 7c). AV-1959R-immune sera bound senile plaques only, AV-1980R-immunie sera bound NFTs and neuritic threads, yet sera from mice immunized with AV-1959R/AV-1980R mixture or AV-1953R bound both pathologies: plaques, neuritic threads and NFTs. Therefore, both mixture of MultiTEP-platform based vaccines and the dual vaccine could be an effective active immunotherapeutic strategy for targeting both misfolded proteins involved in AD pathology.

### Cross synergism in MultiTEP-based vaccines targeting different antigens

**Figure 8:**
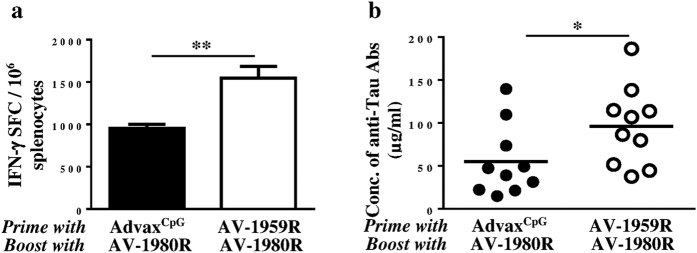
Humoral and cellular immune responses in mice vaccinated twice with AV-1959R and boosted (single boost) with AV-1980R formulated with Advax^CpG^ adjuvant. (**a**) Numbers of IFN-γ producing cells were detected by ELISpot in splenocyte cultures. Bars represent average ± SD for n = 4/per group. (**b**) Concentrations of anti-tau antibodies were measured by ELISA. Lines represent mean values for n = 10/per group (*P < 0.05, **P < 0.01*, t*-test).

All the above vaccines are based on our universal MultiTEP vaccine platform that is based on a string of Th foreign epitopes which, as shown in monkeys, can stimulate immune responses in a broad population of subjects with high MHC class II gene polymorphisms^10^. Notably, the universal MultiTEP platform may allow using two vaccines targeting Aβ and tau at early and late stages of the disease, respectively. At the initiation of anti-tau immunotherapy AD patient immunized previously with anti-Aβ vaccine would have large numbers of MultiTEP-specific memory Th cells and hence will rapidly generate therapeutic concentrations of antibodies. To simulate this situation, we immunized mice with AV-1959R formulated in Advax^CpG^ or injected with Advax^CpG^ only (control) and both groups were boosted with vaccine targeting tau B cell epitope, AV-1980R. Boosting of AV-1959R vaccinated mice with AV-1980R, but not sham-injected mice, induced significantly higher cellular (Fig. 8a) and humoral (Fig. 8b) immune responses, thus proving the synergistic effect of sequential immunization with different MultiTEP vaccines.

## Discussion

AD is the most common cause of dementia, and worldwide nearly 46 million people have Alzheimer’s or related dementias and this number is estimated to increase to 131.5 million by 2050^27^. Currently, the majority of neuroscientists agree that the optimal targets for AD immunotherapy are pathological forms of Aβ and tau. The relationship between these molecules in progression of AD pathology is still controversial, with the majority of data supporting Aβ aggregation being the primary event in AD pathogenesis, secondarily triggering production of pathological tau^28^,^29^,^30^,^31^,^32^,^33^. Nonetheless, accumulation of pathological tau in the brains correlates with dementia in AD patients. Thus, by the time clinical symptoms appear, there is already substantial tau pathology in the brains^34^,^35^. Therefore, we propose that anti-Aβ immunotherapy should be initiated at the early stages of AD to minimize synaptic and neuronal loss, while anti-tau immunotherapy may be most effective when disease progression is more advanced^33^. Thus we have decided to generate vaccines that will target pathological molecules either sequentially (e.g. Aβ vaccinations followed by tau vaccination) or simultaneously (mixture of Aβ plus tau vaccines, or dual-epitope vaccine containing both Aβ and tau B cell epitopes). Importantly, although clinical data from anti-tau immunotherapy are not currently available, results from anti-Aβ active and passive vaccination trials suggest that only relatively high concentrations of antibodies are therapeutically potent^9^,^30^,^31^,^32^,^33^,^36^. Therefore, to enhance immune response in elderly patients with immunosenescence^37^ we have developed recombinant protein-based Aβ and tau, or dual-epitope vaccines by fusing relevant epitopes of these pathological proteins with our MultiTEP platform which is composed of 12 foreign Th cell epitopes, which are the promiscuous PADRE epitope, epitopes from Tetanus Toxin (P23, P32, P21, P30, P2, P7, P17 and P28), Hepatitis B (HBVnc & HBsAg), and influenza virus (MT)^10^. We hypothesized that this should provide a broad coverage of human MHC class II polymorphism utilizing the wide array of tetanus toxin, hepatitis B and influenza Th epitopes incorporated into the MultiTEP platform. We have previously shown that MultiTEP platform-based DNA vaccine targeting Aβ_1-11_ was immunogenic in mice, rabbits, and in monkeys with highly polymorphic MHC class II genes^10^,^11^,^12^,^13^. Here we further suggested that the same MultiTEP platform used for two different recombinant protein-based vaccines targeting Aβ and tau could be beneficial because Th cells activated by AV-1959R will generate strong Th memory cells and will facilitate rapid production of anti-tau antibodies in the future vaccination/s of the same organism with the anti-tau vaccine, AV-1980R. In this study we report for the first time on the immunogenicity of vaccines targeting Aβ, tau or both pathological molecules simultaneously or sequentially.

It is well known that to be immunogenic any recombinant protein should be formulated in a potent adjuvant. In the AN-1792 trials a substantial portion of vaccinated subjects were non-responders, even though the strong QS21 adjuvant was used^31^,^38^. Thus, the first aim of this study was to identify an optimal adjuvant for use in future AD vaccine trials. Figures 1 and 2 clearly demonstrate that Advax^CpG^ formulated with AV-1959R vaccine provided significantly higher Th cell and anti-Aβ antibody responses than any other adjuvant approved for human use or tested in clinical trials. Moreover, Advax^CpG^ enhanced both Th2 and Th1 immune responses as reflected by the highest induction of not only IgG1, but also IgG2a^b^ antibody isotype. Antibodies of the IgG2a^b^ isotype exhibit the highest level of Fc effector function, which may provide the utmost efficiency in AD immunotherapy^39^, and the acute pro-inflammatory responses (IFN-γ^+^SFC) generated by AV-1959R formulated with Advax^CpG^ could be beneficial for AD treatment as well, since TLR4 agonist MPLA reduced Aβ load in the brain of mice and enhanced cognitive function^40^. Finally, MultiTEP-based vaccine design is anticipated to be safe, because Th epitopes in this platform are all foreign and are not expressed in the brain.

While this is the first report of Advax^CpG^ being used for enhancement of the immune response against a B cell self-epitope fused with MultiTEP, the potency of this adjuvant has been previously demonstrated for vaccines targeting viral and bacterial antigens^18^. Importantly, a pandemic influenza vaccine formulated with Advax™ was effective in human subjects up to 90 years of age, enhancing anti-influenza plasmablast and antibody responses across the whole age spectrum^24^. The ability of this delta-inulin based adjuvant to help counter the normal age-related decline in plasmablast and antibody responses to influenza immunization is likely to be important for human AD vaccine development, as these will principally need to be administered to elderly subjects with immuno-senescence^34^.

As mentioned above, the difference in the timing of Aβ and tau accumulation^34^ and their different roles in the onset and progression of AD, suggest that sequential vaccinations with Aβ and tau vaccines, or even combined vaccine against both molecules might represent the most effective AD approach. In particular, the same vaccine platform could be used to both prevent the onset of AD^41^,^42^,^43^,^44^, and also slow down development of tauopathy-associated dementia^32^,^45^,^46^,^47^,^48^,^49^,^50^,^51^. In order to utilize such an approach we have developed three MultiTEP platform-based vaccines targeting Aβ, tau or both molecules simultaneously. All MultiTEP-based vaccines formulated with Advax^CpG^ induce strong cellular and humoral (concentrations of antibody and numbers of ASC) immune responses (Figs 4 and 5). Co-immunizations with AV-1959R and AV-1980R vaccines provided high antibody titers against Aβ and tau epitopes, respectively and these humoral immune responses did not differ from results of immunization with single AV-1980R or AV-1959R vaccines. However, the AV-1953R dual vaccine demonstrated significantly decreased anti-tau humoral immune responses compared with mice immunized with AV-1980R or a mixture of AV-1980R and AV-1959R vaccines (Figs 4 and 5). These decreases in antibody titers and numbers of ASC were not associated with insufficient Th cell stimulation by AV-1953R vs AV-1980R/AV-1959R, since the Th cell responses did not differ significantly between these groups (Fig. 3). This suggested that the three copies of tau_2-18_ B cell epitope in the MultiTEP vaccine might not be adequately presented on the surface of the folded AV-1953R or AV-1980R protein. In the absence of crystal structures, we performed an *in silico* approach to create structural models of the MultiTEP-based proteins that were further optimized using molecular dynamic simulation. From the 3D *in silico* models of these vaccines it appears that tau_2-18_ epitopes may have a less optimal presentation on the surface of AV-1953R dual vaccine than on the AV-1980R single one (Fig. 6). These subtle differences in the structural configuration may have played a critical role in the observed differences in the antibody titers and ASC numbers. Another possible reason could be B cell immunodominance of the Aβ epitope which may suppress the maturation of tau-specific B cells by competition for antigen-specific Th cells. However, this was not the case when immunization was performed with a mixture of the two vaccines containing the same Th epitopes (Fig. 5). In addition, we have performed experiments with increased doses of AV-1953R and did not see an increase of anti-tau antibody concentrations too (data not shown).

Regardless of the exact cause for the varying immunogenicity seen with the different MultiTEP-based tau vaccines it is clear that AV-1980R vaccine was more immunogenic than dual-epitope vaccine, AV-1953R, and therefore, it could be used for AD therapy following preventive vaccinations with AV-1959R. Mice primed by AV-1959R responded significantly better to the booster vaccination with AV-1980R (Fig. 8a,b). Importantly, regardless of the vaccine used (single, mixture or dual), the generated antibodies are potentially functional and bind equally well to the pathological deposits of Aβ and tau in the brain sections from four AD cases (Fig. 7). It is important to note, that antibodies were effectively binding oligomeric forms of Aβ and tau, which are widely believed to be much more toxic than the fibrils and plaques^52^.

These data may open a new avenue for future vaccination regimens, as the preventive AV-1959R could be used in prodromal AD subjects and then, in case of subsequent disease progression, boosting could be performed with the AV-1980R vaccine. Hence, using AV-1959R as a preventive vaccine, and then adding AV-1980R after onset of AD may provide the broadest protection across the AD clinical disease spectra.

In summary, optimal AD vaccine formulation, adjuvant selection and targeting of the right epitopes at the appropriate stage of disease will be crucial to a successful immunotherapeutic approach. An AD vaccine based on target epitopes attached to universal, highly immunogenic MultiTEP vaccine platform when combined with Advax^CpG^, a potent yet well tolerated adjuvant suitable for human use, induced extremely high antibody titers against both Aβ and tau, at levels not previously achieved in other studies. The MultiTEP strategy provides a unique opportunity to generate high antibody responses in subjects by utilizing memory Th cells previously generated in the human population in response to infection or vaccination by tetanus toxin, hepatitis B and influenza. The MultiTEP platform may be especially beneficial in the elderly because the vaccine utilizes memory T cells that are in abundance in elderly patients instead of relying on activation of naïve T cells, which decline with age. Therefore, the universal and highly immunogenic MultiTEP vaccine platform shows promise for both preventive and therapeutic approaches in AD.

## Methods

### Mice

Female, 6–8 weeks old C57BL/6 mice (H-2^b^ haplotype) were obtained from Jackson Laboratory. All animals were housed in a temperature and light-cycle controlled facility, and their care was under the guidelines of the NIH and an approved IACUC protocol at UC Irvine. Animal experiments were performed according to the guideline of the Animal Care and Use Committee of UCI and approved by University Laboratory Animal Resources (ULAR).

### Epitope vaccines

To prepare three recombinant proteins, minigenes encoding 3Aβ_1-11_-MultiTEP, 3Tau_2-18_-MultiTEP or 3Aβ_1-11_-MultiTEP-3Tau_2-18_ were cloned into the modified *E. coli* expression vector pET11 (for AV-1959R; Novagen, MA) or pET24a (for AV-1980R and AV-1953R; Novagen, MA) in frame with 6xHis-Tag at the C-terminus. DNA sequencing was performed to confirm that the generated plasmids contained the correct sequences.

### Purification of proteins

Recombinant proteins were purified from *E. coli* BL21 (DE3) cells transformed with pET11/3Aβ_1-11_-MultiTEP, pET24a/3Tau_2-18_-MultiTEP or pET24a/3Aβ_1-11_-MultiTEP-3Tau_2-18_ plasmids as described^13^,^53^. The final recombinant protein was analyzed in 10% Bis-Tris gel electrophoresis (NuPAGE Novex Gel, Invitrogen, CA). Protein bands were visualized by Coomassie dye (Fig. S2a) and specificity of the bands was confirmed by Western Blot (WB) using 6E10 (Fig. S2b) and anti-tau_2-18_ 1C9 (Fig. S2c) monoclonal antibodies. The level of endotoxin was measured using E-TOXATE kits, as recommended by the manufacturer (Sigma, St Louis, MO).

### Experimental protocols

#### Study 1. Selection of an optimal adjuvant

Seven groups of C57BL/6 mice (n = 5–8) were immunized with 20 μg of AV-1959R (pET11/3Aβ_1-11_-MultiTEP) formulated with a selection of adjuvants: Montanide-*ISA51* (s.c.; 50/50 ratio; SEPPIC, France), Montanide-*ISA720* (s.c.; 50/50 ratio; SEPPIC, France), MPLA-SM (s.c.; 10 μg; Enzo, NY), Alhydrogel^®^ (s.c.; 70 μg; BRENNTAG, Denmark), Quil-A (s.c.; 20 μg; BRENNTAG, Denmark), Advax™, and Advax^CpG^ (both i.m.; 1mg; Vaxine Pty Ltd, Adelaide, Australia). All mice were injected four times at biweekly intervals. Sera were collected 14 days after the third immunization and were used to measure anti-Aβ antibody responses. On day 7 after the last injection mice were terminated and cellular immune responses were analyzed in splenocytes.

#### Study 2. Efficacy of different AD vaccines

Four groups of C57BL/6 mice (n = 8 per group) were immunized with AV-1959R (20 μg/per mouse/per injection), AV-1980R (pET24a/3Tau_2-18_-MultiTEP, 20 μg/per mouse/per injection), AV-1953R (pET24a/3Aβ_1-11_-MultiTEP-3Tau_2-18_, 20 μg/per mouse/per injection) and mixture of AV-1959R and AV-1980R proteins (20 μg protein/mouse/injection), all formulated with Advax^CpG^ adjuvant (1 mg/mouse/injection). Control groups of mice were injected with Advax^CpG^ adjuvant only or PBS (n = 6 for both groups). All mice were injected four times at biweekly intervals. Sera were collected 14 days after third immunizations and anti-Aβ and anti-tau antibody responses were analyzed. On day 7 after the last injection mice were terminated and cellular immune responses were analyzed in splenocytes.

#### Study 3. Cross synergism in MultiTEP-based vaccines

We studied the effect of switching from AV-1959R to AV-1980R. Two groups of mice (n = 14) were immunized twice with AV-1959R formulated with Advax^CpG^ or injected with Advax^CpG^ only. Then both groups received a single vaccination with AV-1980R formulated with Advax^CpG^. Four mice from each group were terminated at day 7 after the third injection and T cell responses were measured in splenocytes. The remaining mice (n = 10 per group) were bled at day 14 after immunization for measurement of serum antibody responses.

### Detection of Aβ- and tau-specific antibodies

The concentrations of anti-Aβ and anti-tau antibodies in serum were determined by ELISA, as described previously^11^,^53^,^54^. Anti-Aβ and anti-tau antibody concentrations were calculated using a calibration curve generated with 6E10 (Covance, CA) and 1C9 mAbs (generated at the Institute for Molecular Medicine, Huntington Beach, CA), respectively. HRP-conjugated anti-mouse IgG (Jackson ImmunoResearch Laboratories, ME) was used as secondary antibody. HRP-conjugated anti-IgG1, IgG2a^b^, IgG2b and IgM specific antibodies (Bethyl Laboratories, Inc.) were used to characterize the isotype profiles of anti-Aβ antibodies in individual sera at different dilutions (see legend of Fig. S1).

### Proliferation of splenocytes and detection of splenocytes producing IFN-γ and IL-4

Analysis of IFN-γ and IL-4 producing T cells was performed in splenocyte cultures from immunized mice by ELISpot assay (BD Biosciences, CA), as previously described^10^,^44^,^55^,^56^. Cultures of splenocytes were re-stimulated *in vitro* with a cocktail of 12 peptides representing the Th epitopes in the MultiTEP vaccine^10^ (2 μg/ml of each peptide), soluble Aβ_40_, tau_2-18_ or irrelevant peptides at 10 μg/ml for 20 hours. The numbers of SFC per 10^6^ splenocytes stimulated with Th peptides or Aβ_40_ or tau_2-18_, were then counted.

T cell proliferation assay was performed in splenocyte cultures using a [^3^H]-thymidine incorporation assay, as previously described^53^,^57^. The cultures of splenocytes from mice were re-stimulated *in vitro* with the same antigens that were used in ELISpot assay. Stimulation index (SI) was calculated for each mouse using the formula: SI = experimental count/spontaneous count, where spontaneous count wells included irrelevant peptide.

### Detection of B cells producing Aβ- and tau-specific antibodies

Antibody-secreting B cells (ASC) specific to Aβ and tau were detected in splenocytes by ELISpot (Mabtech Inc, Cincinnati, OH). Splenocytes from experimental and control mice were incubated for 24 hours in 96-well plates coated with Aβ_42_ or tau_2-18_ peptides. After incubation the assay was performed as recommended by the manufacturer (Mabtech Inc, Cincinnati, OH).

### Preparation of brain homogenates and western blot analysis

#### Soluble and insoluble fractions

0.2 g of brain tissue from four different AD cases were homogenized in 0.4 ml TBS buffer with Halt™ Protease and Phosphatase Inhibitor Cocktail (100X, Thermo Scientific, CA), then centrifuged at 6400xg for 15 minutes at +4 °C. Supernatants were collected and stored at −80 °C for further analysis as soluble fractions. Remaining pellets were solubilized in 70% formic acid (FA) and centrifuged at 100,000 × g for 1 hr at +4 °C. Avoiding upper lipid layer, the lower aqueous layer was collected and stored at −70 °C for later quantification of insoluble Aβ and tau levels. Prior to the next step FA fractions were neutralized with 5N sodium hydroxide (NaOH) following a neutralization buffer (1 M TRIS Base, 0.5 M NaH_2_PO_4_) and precipitated with ice cold 10% Trichloroacetic acid (TCA) in acetone. Then precipitated fractions were resolubilized in equal volume of TBS. Protein concentration was determined using BCA Protein Assay Kit (Pierce, IL) and samples were adjusted to the equal concentration.

#### Western Blots

WB with soluble and insoluble fractions of four brain homogenates were performed as described in^10^,^44^,^55^,^56^. Briefly, soluble and insoluble fractions applied to electrophoresis on NuPAGE 4–12% Bis-Tris gel in MES buffer under reducing conditions (Invitrogen, CA) and electrotransferred onto nitrocellulose membrane (GE Healthcare, NJ). For antigen retrieval, membranes with soluble fractions were boiled in PBS at 100 °C as described in Rosen *et al.*^58^ and then were blocked with 5% fat-free dry milk. Membranes with insoluble fractions were blocked without antigen retrieval. Aβ and tau were visualized by incubating with sera from mice immunized with AV-1959R, AV-1980R, AV-1953R or mixture of AV-1959R and AV-1980R followed by HRP-conjugated anti-mouse IgG (Santa Cruz Biotechnology, CA). Sera were used after normalization of antibody concentration measured by ELISA (1 μg/ml for anti-Aβ and 0.4 μg/ml for anti-tau).

### Detection of Aβ plaques and tau tangles in human brain tissues by IHC

Sera from mice immunized with AV-1959R, AV-1980R, AV-1953R and mixture of AV-1959R and AV-1980R were screened for the ability to bind to human Aβ plaques or/and tau tangles using 40 μm brain sections of formalin-fixed cortical tissues from four different severe AD cases (received from Brain Bank and Tissue Repository, MIND, UC Irvine) using immunohistochemistry as described previously^10^,^44^,^55^,^56^. A digital camera (Olympus, Tokyo, Japan) was used to capture images of the plaques at 60× magnification.

### Construction of molecular models

Sequences corresponding to each individual epitope within each MultiTEP protein (AV-1980R, AV-1959R AV-1953R) were submitted to the PSIPRED protein structure prediction server^59^ for folding profile based threading. Those template structures matching the folding pattern of the query with <=0.0001 *p*-value were selected (The individual templates used and the appropriate references are provided in Supplementary Tables S1–S3). Based on the multiple sequence alignment between the query and the templates, homology models were generated using the homology modeling program Modeller v9.13 60. Then each homology model was evaluated, refined and selected based on the discrete optimized protein energy score (DOPES). Then models were relaxed using the molecular dynamics simulation program NAMD v2.9 61 for 1000 steps at 310 K temperature. Finally, models of the synthetic MultiTEP proteins containing Aβ_1-11_ or tau_2-8_ epitopes and the combination were visualized using the molecular modeling program UCSF chimera^62^.

### Statistical analysis

Statistical parameters (mean, standard deviation (SD), significant difference, etc.) were calculated using the Prism 6 software (GraphPad Software, Inc., La Jolla, CA). Statistically significant differences were examined using a *t-test* or analysis of variance (ANOVA) and Tukey’s multiple comparisons post-test (a P value of less than 0.05 was considered significant).

## Additional Information

**How to cite this article**: Davtyan, H. *et al.* Alzheimer’s disease Advax^CpG^- adjuvanted MultiTEP-based dual and single vaccines induce high-titer antibodies against various forms of tau and Aβ pathological molecules. *Sci. Rep.* **6**, 28912; doi: 10.1038/srep28912 (2016).

## Supplementary Material

Supplementary Information
