# Supplementary material for: Alzheimer’s disease AdvaxCpG- adjuvanted MultiTEP-based dual and single vaccines induce high-titer antibodies against various forms of tau and Aβ pathological molecules
Source: Sci Rep. 2016 Jul 1;6:28912. doi: 10.1038/srep28912 (PMC4929459; doi:10.1038/srep28912)
Supplement: Supplementary Information [file srep28912-s1.pdf]

Supplementary Information

Alzheimer's disease Advax^{CpG}- adjuvanted MultiTEP-based dual and single vaccines induce high-titer antibodies against tau and Aβ

Hayk Davtyan, Karen Zagorski, Harinda Rajapaksha, Armine Hovakimyan, Arpine Davtyan,

Irina Petrushina, Konstantin Kazarian, David H. Cribbs, Nikolai Petrovsky, Michael G.

Agadjanyan, Anahit Ghochikyan

Figure S1

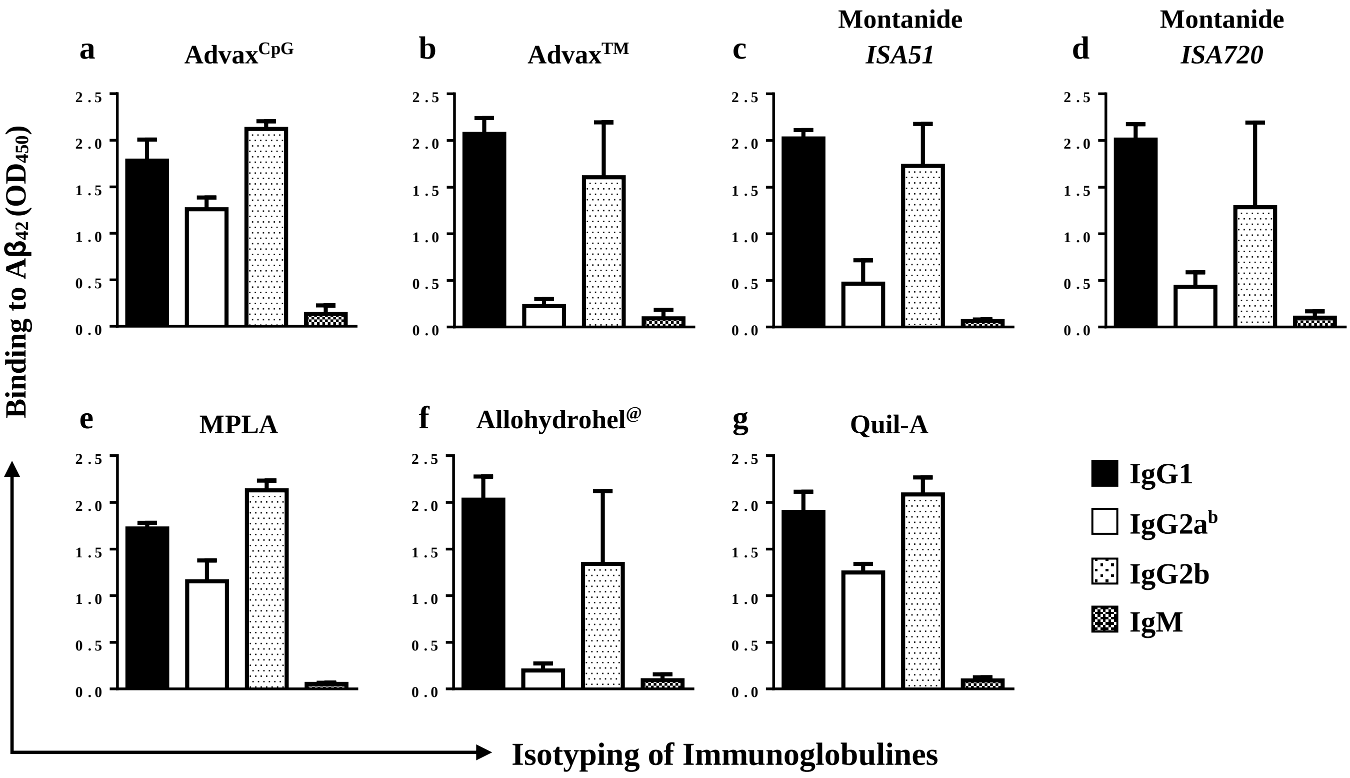

Anti-Aβ IgG1, IgG2a^{b}, IgG2b and IgM antibody responses in mice immunized with AV-1959R formulated with different adjuvants. To normalize ELISA protocol sera from mice were used at different dilutions in each group.

(**a**) AV-1959R+Advax^{CpG} (dilution 1:1000); (**b**) AV-1959R+Advax™ (dilution 1:200); (**c**) AV-1959R+ Montanide-*ISA51* (dilution 1:500); (**d**) AV-1959R+Montanide-*ISA720* (dilution 1:200); (**e**) AV-1959R+MPLA (dilution 1:500); (**f**) AV-1959R+Allohydrohel@ (dilution 1:50); and (**g**) AV-1959R+Quil-A (dilution 1:500). Bars represent average ± SD (n=5 per group).

Figure S2

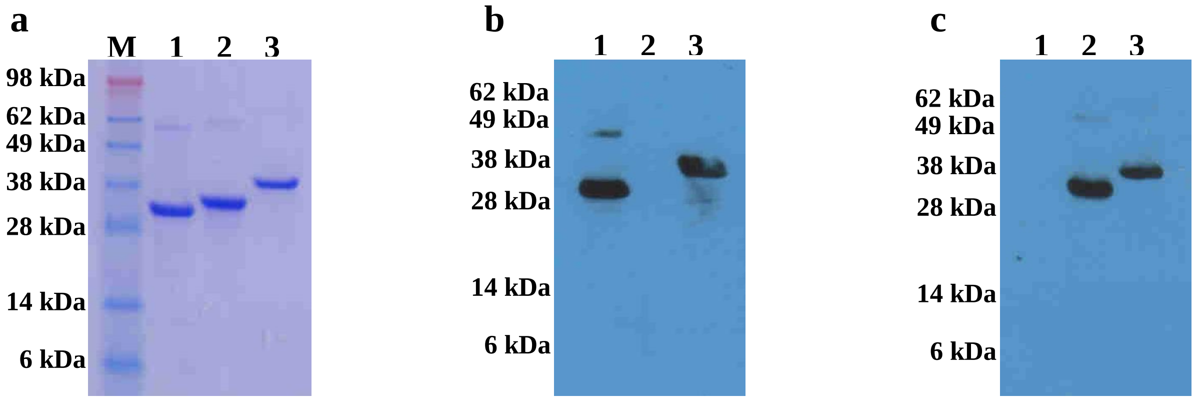

Generation and characterization of recombinant proteins: AV-1959R, AV-1980R and AV-1953R. (**a**) The purity and integrity of all three proteins isolated from *E.coli* was analyzed in a 10% Bis-Tris polyacrylamide gel in MES buffer after visualization by Coomassie dye. The molecular weights of protein bands are corresponded to the theoretically calculated molecular weights (M-Molecular weight marker; Lane 1 - AV-1959R [32.3 kDa], Lane 2 – AV-1980R [33.8 kDa], Lane 3 – AV-1953R [38.0 kDa]). As expected dual vaccine AV-1953R containing both Aβ and tau B cell epitopes is heavier than each single vaccine (AV-1959R and AV-1980R), containing either Aβ or tau B cell epitope. In all vaccines B cell epitopes are attached to the same MultiTEP platform. (**b, c**) The specificity of the bands was confirmed by WB using 6E10 (anti-Aβ; **b**) and 1C9 (anti-Tau; **c**) monoclonal antibodies followed by HRP conjugated anti-mouse IgG (Lane 1 - AV-1959R, Lane 2 – AV-1980R, Lane 3 – AV-1953R).

Table S1. Templates used for AV-1959R

PDB ID	Reference
2NYYA	[3]
3J2VC	[4]
1QGTA	[5]
1F31A	[6]
2G33C	[8]
3RSJA	[7]
3KXSF	[11]

Table S2. Templates used for AV-1980R

PDB ID	Reference
1G55A	[1]
2VU9A	[2]
2NYYA	[3]
3J2VC	[4]
1F31A	[6]
1QGTA	[5]
3RSJA	[7]
3KXSF	[11]

Table S3. Templates used for AV-1953R

PDB ID	Reference
2NYYA	[3]
3J2VC	[4]
1QGTA	[5]
3RSJA	[7]
2G33C	[8]
1UTEA	[9]
3FFZA	[10]
1K6WA	[12]
3V6ZE	[13]

References for Supplementary Material

1 Dong, A. *et al.* Structure of human DNMT2, an enigmatic DNA methyltransferase homolog that displays denaturant-resistant binding to DNA. *Nucleic Acids Res* **29**, 439-448 (2001).

2 Stenmark, P., Dupuy, J., Imamura, A., Kiso, M. & Stevens, R. C. Crystal structure of botulinum neurotoxin type A in complex with the cell surface co-receptor GT1b-insight into the toxin-neuron interaction. *PLoS Pathog* **4**, e1000129 (2008).

3 Garcia-Rodriguez, C. *et al.* Molecular evolution of antibody cross-reactivity for two subtypes of type A botulinum neurotoxin. *Nat Biotechnol* **25**, 107-116 (2007).

4 Yu, X., Jin, L., Jih, J., Shih, C. & Zhou, Z. H. 3.5A cryoEM structure of hepatitis B virus core assembled from full-length core protein. *PLoS One* **8**, e69729 (2013).

5 Wynne, S. A., Crowther, R. A. & Leslie, A. G. The crystal structure of the human hepatitis B virus capsid. *Mol Cell* **3**, 771-780 (1999).

6 Swaminathan, S. & Eswaramoorthy, S. Structural analysis of the catalytic and binding sites of Clostridium botulinum neurotoxin B. *Nat Struct Biol* **7**, 693-699 (2000).

7 Benson, M. A., Fu, Z., Kim, J. J. & Baldwin, M. R. Unique ganglioside recognition strategies for clostridial neurotoxins. *J Biol Chem* **286**, 34015-34022 (2011).

8 Bourne, C. R., Finn, M. G. & Zlotnick, A. Global structural changes in hepatitis B virus capsids induced by the assembly effector HAP1. *J Virol* **80**, 11055-11061 (2006).

9 Guddat, L. W. *et al.* Crystal structure of mammalian purple acid phosphatase. *Structure* **7**, 757-767 (1999).

10 Kumaran, D. *et al.* Domain organization in Clostridium botulinum neurotoxin type E is unique: its implication in faster translocation. *J Mol Biol* **386**, 233-245 (2009).

11 Packianathan, C., Katen, S. P., Dann, C. E., 3rd & Zlotnick, A. Conformational changes in the hepatitis B virus core protein are consistent with a role for allostery in virus assembly. *J Virol* **84**, 1607-1615 (2010).

12 Ireton, G. C., McDermott, G., Black, M. E. & Stoddard, B. L. The structure of Escherichia coli cytosine deaminase. *J Mol Biol* **315**, 687-697 (2002).

13 DiMattia, M. A. *et al.* Antigenic switching of hepatitis B virus by alternative dimerization of the capsid protein. *Structure* **21**, 133-142 (2013).
